# The Necessity for Polypharmacological Research – An Editorial on ‘Network Polypharmacology of ATP-binding Cassette (ABC) and Solute Carrier (SLC) Transporters’

**DOI:** 10.3389/fphar.2025.1561247

**Published:** 2025-03-21

**Authors:** Muhammad Rafehi, Kapil Juvale, Lukasz Pulaski, Sven Marcel Stefan

**Affiliations:** ^1^ University Hospital of Augsburg, Augsburg, Germany; ^2^ Department of Medical Education Augsburg, University of Augsburg, Augsburg, Germany; ^3^ Institute of Clinical Pharmacology, University Medical Center Göttingen, Göttingen, Germany; ^4^ Shobhaben Pratapbhai Patel School of Pharmacy & Technology Management, SVKM’s NMIMS, Mumbai, India; ^5^ Department of Oncobiology and Epigenetics, Faculty of Biology and Environmental Protection, University of Lodz, Lodz, Poland; ^6^ Laboratory of Transcriptional Regulation, Institute of Medical Biology PAS, Lodz, Poland; ^7^ Medicinal Chemistry and Systems Polypharmacology, Lübeck Institute of Experimental Dermatology, University of Lübeck and University Medical Center Schleswig-Holstein, Lübeck, Germany; ^8^ Department of Biopharmacy, Medical University of Lublin, Lublin, Poland

**Keywords:** polypharmacology, multitargeticity, multitarget binding site, polyspecificity, privileged ligands, polysubstrates, drug discovery, drug profiling

Polypharmacology is widely perceived as promiscuity of drugs, i.e., their propensity to address off-targets and thereby cause adverse effects. Multitargeticity of drugs is usually undesired, and thus, mostly inappropriately assessed, described, and reported (Stefan and Rafehi, 2024). The lack of research in this field stands in the way of a deeper understanding of polypharmacology – and the tremendous potential for research and therapy it bears. This Research Topic was edited by researchers working in this field to highlight important aspects of polypharmacology on multiple levels, with a focus on membrane transporters as a prototypical protein group.

A clear and precise language is fundamental for understanding the concept of polypharmacology. Stefan and Rafehi thus provided a first-in-field glossary describing relevant terminology in this field within the broader context of drug discovery and development (Stefan and Rafehi). The interconnection of terminology used in different research areas supports interdisciplinary collaboration, which is essential for broad-scale assessments of bioactivities to characterize the polypharmacology of drugs. Thus, by providing a guideline for the use of correct and inclusive language, Stefan and Rafehi offer a framework for sustainable and collaborative large-scale assessment platforms, sharing data, and high-quality research output.

The conceptualization, development, and validation of new assessment platforms to identify and validate novel potential drug targets requires a repertoire of tools. Here, Song et al. described an easily applicable and transferable LC-MS/MS-based workflow for the assessment of membrane transporter function (Song et al.). This is essential in broad-scale assessments of drug properties and the accurate description and interpretation of polypharmacology in the context of drug safety.

The polypharmacology of drugs depends on several factors, e.g., stereoselectivity toward chiral drugs, like verapamil, that bridge the concepts of polypharmacology and stereoselectivity. Also, the distribution of chemical patterns (Stefan et al., 2022; Namasivayam et al., 2022; Namasivayam et al., 2021a; Namasivayam et al., 2021b) and the resultant physicochemistry (Namasivay et al., 2022) determine the interactions with various targets. On the other hand, the tendency of certain targets to accept multiple ligands (i.e., polyspecificity) depends on structural specificities, e.g., polymorphisms or (drug-selected) mutations, that may be the basis of drug cross-resistance and therapy failure in the case of efflux transporters. Gupta et al. analyzed polyspecific transporters for frequently occurring mutations and their impact on drug binding (Gupta et al.). As it turned out, the binding of polypharmacological transporter substrates (“polysubstrates”), e.g., mitoxantrone, is critically influenced by key mutations–suggesting the existence of “backbone amino acids” that constitute polyspecificity (i.e., “multitarget binding sites”) (Stefan et al., 2025; Namasivayam et al., 2021c).

Concerning structural commonalities of proteins that are otherwise functionally and phylogenetically distant from each other, “translation between species” is an important field to understand differences between species and to project this knowledge to useful research applications. Luckenbach and Burkhardt-Medicke emphasized this topic by studying human and zebrafish transporters, revealing that the operational temperature needs to vary due to the homoio- and poikilotherm nature of the respective organisms (Luckenbach and Burkhardt-Medicke). However, although (related) proteins of different species work at different operational conditions, polypharmacologicals such as verapamil still exert their effects. The findings of Luckenbach and Burkhardt-Medicke thereby contribute to a better understanding of polypharmacology.

The regulation of protein expression is as important for their function as for potential genetic and structural changes. It could not only be influenced by polypharmacologicals themselves, but it could also change the pharmacology of polypharmacologicals due to an altered target or modifier expression. Puris et al. have focused on such expression differences for membrane transporters in female and male mice applying polyanalytical approaches (Puris et al.). Sex inequality in medical research–and its relevance for the polypharmacology of drugs–is still a regularly neglected issue that bears particular relevance for disease treatment and equality in public health services and advanced therapies.

In this context, polypharmacy, i.e., therapy with multiple drugs, has a solid standing in both classical pharmacotherapy and traditional Chinese medicine. This is particularly true considering the network polypharmacology of multitarget drugs, and their ability to address a variety of structurally, functionally, and/or phylogenetically distant proteins, e.g., membrane transporters, ion channels, or kinases. Wang et al. addressed this aspect by identifying the bioactive constituents of a traditional Chinese herbal formula, Qingdu Fang, by polyanalytical and bioinformatic approaches (Wang et al.). Amongst the identified active ingredients was quercetin, a flavonoid known for its very rich polypharmacology–above all regarding membrane transporters–that indeed represents a useful template for addressing new targets and target networks.

Despite its potential, polypharmacology is regularly disregarded as an independent (and important) research field:

(i) It is being actively denied that the polypharmacology of drugs is used for medical purposes, claiming that academic and commercial research is not focusing on it as a strategy to improve therapeutic benefit. This is certainly untrue, as many drugs are (a) still widely used despite their lack of specificity [e.g., tyrosine kinase inhibitors (TKIs) or central nervous system (CNS) drugs], (b) re-purposed to tackle prevalent and orphan diseases (e.g., acetylsalicylic acid, minoxidil, sildenafil, thalidomide, etc.), (c) intentionally developed to engage multiple targets (e.g., tirzepatide), or (d) used in a drug combination therapy; collectively also addressing multiple targets (e.g., cancer, HIV infection, tuberculosis, etc.) (Rafehi et al., 2024);
(ii) It is regularly argued that “specificity is key”. However, any research in this field to understand (and ultimately prevent) polypharmacology of drugs for improving drug specificity and safety is discouraged, eventually resulting in the opposite: insufficiently characterized, and thus, ultimately less safe drugs;
(iii) It is certainly true that initially discovered and/or deliberately designed polypharmacologicals may bear affinities for the desired (and potentially also undesired) targets that are either too similar or too different to each other, leading to off-target effects and adverse events. However, active research is necessary to optimize such drugs and balance their activities, which also requires the support and endorsement of scientific communities;
(iv) Indeed, drug and target repurposing programs are particularly tedious, as initial screenings against a panel of targets are necessary. However, by actively supporting such programs, global research as a whole could be strengthened, allowing local researchers to grow their expertise rather than acquiring all expertise within a single (financially well-equipped) laboratory which would ultimately undermine all efforts toward collaboration, green science, sustainability, and equality of research;
(v) A thorough understanding of polypharmacology and polyspecificity could provide a basis for the druggability of as yet undruggable targets (Stefan and Rafehi, 2023). As the possibilities for the arrangement of amino acids within proteins is limited and common structural motifs have been described earlier (i.e., “superfolds,” “supersites,” and “multitarget binding sites”) (Stefan et al., 2025; Namasivayam et al., 2021c), multitarget drugs (“privileged ligands”) with specific chemical entities showing activities against various structurally, functionally, and/or phylogenetically distinct proteins could represent particularly valuable tools for target validation and drug discovery (Stefan et al., 2022; Namasivayam et al., 2022; Namasivayam et al., 2021a; Namasivayam et al., 2021b; Namasivay et al., 2022). However, since the initial screening compounds are perceived in most cases to have rather low potency, many researchers believe that there is no interest in weak starting points for further hit-to-lead optimization. Nevertheless, previous studies have shown that initial screenings with polypharmacologicals may result in very potent and promising hit molecules (Haupenthal et al., 2024). And even if their activity is not favorable: *one* starting point is always better than *none*;
(vi) Even the “undruggability” of newly discovered targets is often denied. It is claimed by some to be a surrogate for low interest in the respective target, issues with assay development of the target, or lack of resources or effort from the scientific communities–thereby questioning the need for the development of privileged ligands at all. However, it is specifically these reasons that can also be used to justify the development of privileged ligands: Undoubtedly, the medicinal chemistry repertoire is limited and currently unable to serve particular fields to explore certain “undruggable” targets. Once properly identified, assessed, and reported, privileged ligands are inexpensive, easily accessible, and sustainable tools that may enrich the pool of methodologies in drug development and medical life sciences.

In conclusion, active research in polypharmacology matters–be it deliberately designing multitarget ligands or specific drugs. There is much more to the concept of polypharmacology than just “dirty drugs,” “frequent hitters,” “bad actors”, and “nuisance compounds” – it is more about their *potential* rather than their *risks*. All contributors of this Research Topic provided important puzzle pieces on various aspects of polypharmacology that improve our collective understanding and ultimately support the development of beneficial, safe, and sustainable drugs.
